# Lung Injury Risk in Traumatic Brain Injury Managed With Optimal Cerebral Perfusion Pressure Guided-Therapy

**DOI:** 10.2478/jccm-2023-0009

**Published:** 2023-05-08

**Authors:** Celeste Dias, Alexandre de Castro, Rita Gaio, Ricardo Silva, Eduarda Pereira, Elisabete Monteiro

**Affiliations:** 1 Faculty of Medicine, University of Porto, Porto, Portugal; 2 Faculty of Mathematics, University of Porto, Porto, Portugal; 3 Centre of Mathematics of the University of Porto, Porto, Portugal; 4 University Hospital Centre São João, Porto Portugal

**Keywords:** traumatic brain injury, acute lung injury, autoregulation, optimal cerebral perfusion pressure, pressure reactivity index, driving pressure, ARDS

## Abstract

**Introduction:**

Management of traumatic brain injury (TBI) has to counterbalance prevention of secondary brain injury without systemic complications, namely lung injury. The potential risk of developing acute respiratory distress syndrome (ARDS) leads to therapeutic decisions such as fluid balance restriction, high PEEP and other lung protective measures, that may conflict with neurologic outcome. In fact, low cerebral perfusion pressure (CPP) may induce secondary ischemic injury and mortality, but disproportionate high CPP may also increase morbidity and worse lung compliance and hypoxia with the risk of developing ARDS and fatal outcome. The evaluation of cerebral autoregulation at bedside and individualized (optimal CPP) CPPopt-guided therapy, may not only be a relevant measure to protect the brain, but also a safe measure to avoid systemic complications.

**Aim of the study:**

We aimed to study the safety of CPPopt-guided-therapy and the risk of secondary lung injury association with bad outcome.

**Methods and results:**

Single-center retrospective analysis of 92 severe TBI patients admitted to the Neurocritical Care Unit managed with CPPopt-guided-therapy by PRx (pressure reactivity index). During the first 10 days, we collected data from blood gas, ventilation and brain variables. Evolution along time was analyzed using linear mixed-effects regression models. 86% were male with mean age 53±21 years. 49% presented multiple trauma and 21% thoracic trauma. At hospital admission, median GCS was 7 and after 3-months GOS was 3. Monitoring data was CPP 86±7mmHg, CPP-CPPopt -2.8±10.2mmHg and PRx 0.03±0.19. The average PFratio (PaO_2_/FiO_2_) was 305±88 and driving pressure 15.9±3.5cmH_2_O. PFratio exhibited a significant quadratic dependence across time and PRx and driving pressure presented significant negative association with PFRatio. CPP and CPPopt did not present significant effect on PFratio (p=0.533; p=0.556). A significant positive association between outcome and the difference CPP-CPPopt was found.

**Conclusion:**

Management of TBI using CPPopt-guided-therapy was associated with better outcome and seems to be safe regarding the development of secondary lung injury.

## INTRODUCTION

Management of severe Traumatic Brain Injury (TBI) remains a conflicting issue in neurocritical care. The greatest difficulty in managing acute TBI patients is to avoid secondary ischemic brain injuries without developing systemic complications [1], namely lung injury and acute respiratory distress syndrome (ARDS) [2–5]. Patients with severe TBI are at risk of developing ARDS which is recognized as one of the significant contributors to mortality in the ICU. Various factors seem to be involved in the pathophysiology of lung injury associated to acute brain injury, including inflammation, altered neurotransmission, hypoxemia and adverse events of mechanical ventilation [6].

After the first definition in 1967, ARDS has been a mutable concept and multiple definitions were proposed. In 2012, Berlin Definition was developed and defined ARDS as an acute diffuse, inflammatory lung injury, which leads to an impairment of pulmonary function within oxygenation compromise and occurring within one week of a known clinical insult. It was proposed a 3 mutually exclusive categories of ARDS: mild, moderate and severe based on the degree of hypoxemia evaluated with PFratio (PaO_2_/FIO_2_) [7]. The potential risk of developing ARDS leads to therapeutic decisions such as fluid balance restriction, high PEEP and other lung protective measures, that may conflict with neurologic outcome [3]. In fact, low cerebral perfusion pressure (CPP) may induce secondary ischemic injury and mortality, but disproportionate high CPP may also increase morbidity and worse lung compliance and hypoxia with the risk of developing ARDS and fatal outcome [8–10].

Since 2017, the most recent published Guidelines of the Brain Trauma Foundation (BTF) recommend to target CPP between 60 and 70mmHg (level IIB) [11]. According to these Guidelines, the main targets in TBI population are to avoid hypoxia and cerebral hypoperfusion, which may be achieved by the optimization of PEEP and CPP to promote both lung recruitment with normoxia and adequate cerebral perfusion [11]. However, the use of PEEP may be controversial in TBI patients because of the reduction of cerebral venous return with potencial increase in ICP and compromise of CPP [5]. Nonetheless, considerable uncertainty still exists about the optimal level of PEEP and CPP and even the BTF Guidelines stress that minimum CPP threshold may depend on the autoregulatory status of individual patients [11,12]. Nowadays, there is accumulated evidence that continuous monitoring of cerebral autoregulation based on evaluation of cerebrovascular pressure reactivity with pressure reactivity index (PRx) has allowed to estimate individualized optimal CPP (CPPopt) at bedside and this approach has been related to better outcome after severe TBI [13–19].

**Fig. 1.**
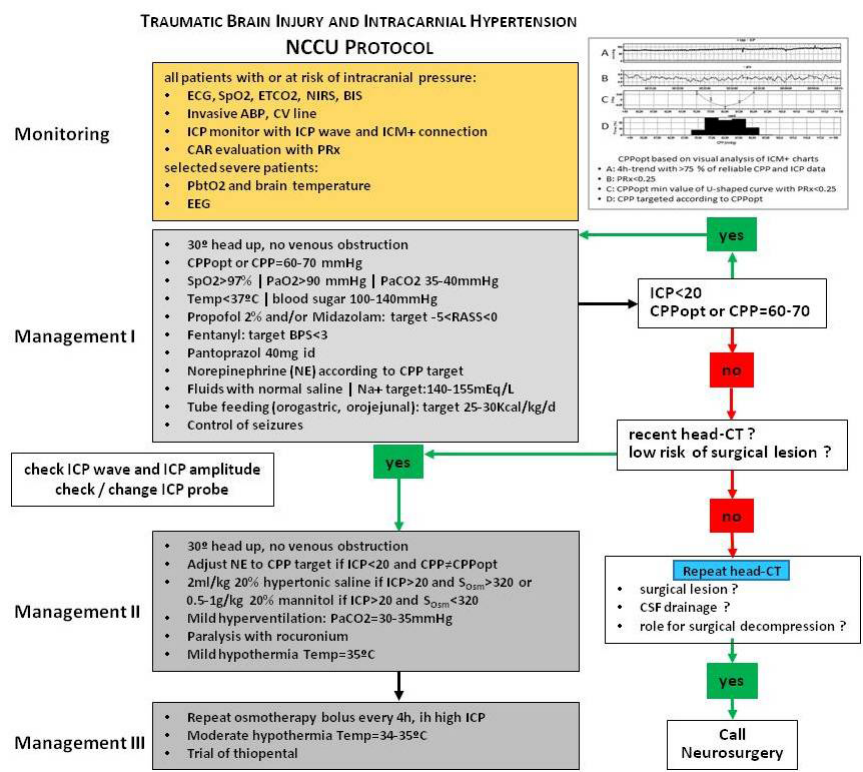
Neurocritical Care Unit (NCCU) protocol for Traumatic Brain Injury and Intracranial Hypertension Management. Optimal cerebral perfusion pressure evaluated continuously at bedside with cerebrovascular reactivity index and intracranial pressure control below 20 mmHg are primary targets. ECG electrocardiogram, SpO_2_ pulse oximetry, ETCO_2_ endtidal carbon dioxide, ABP arterial blood pressure, NIRS cerebral oximetry with near-infrared light, BIS bispectral index, CVP central venous pressure, ICP intracranial pressure, ICM+ multimodal brain monitoring software, CAR cerebral autoregulation, PRx cerebrovascular reactivity index CPP cerebral perfusion pressure, CPPopt optimal CPP, PbtO_2_ brain tissue oxygen pressure, EEG electroencephalogram, PaO_2_ arterial oxygen pressure, PaCO_2_ arterial carbon dioxide pressure, Temp temperature, RASS Richmond agitation–sedation scale, BPS behavioral pain scale, Na+ serum sodium, SOsm serum osmolalityhead-CT head computerized tomography, CSF cerebral spinal fluid.

In our Neurocritical Care Unit (NCCU) in Porto, patients with severe TBI are treated with a CPPopt-guided therapy protocol according cerebral autoregulation evaluation at the bedside using PRx (Figure 1) [20]. Frequently, CPPopt is higher than CPP recommended by BTF Guidelines and therefore, the natural concern arose to validate the safety of this protocol. A previous unpowered published pilot study of 30 patients admitted to the NCCU with TBI showed that CPPopt may not increase the risk of lung injury [20].

The aim of this single center study was to analyze if severe TBI patients admitted to the NCCU and managed with CPPopt-guided protocol had increased risk of PFratio deterioration, lung injury or ARDS and therefore associated worse outcome.

## MATERIALS AND METHODS

### Patient selection

We performed a retrospective analysis of prospective recorded data of all trauma patients, including multiple trauma, with severe TBI admitted to the NCCU between July 2011 and June 2017. Patients were managed with CPPopt-guided protocol using PRx calculated with ICM+® software developed by Cambridge University [17].

All patients needed sedation and ventilation according to NCCU protocol (Figure 1) related to the initial severity or early cerebral deterioration. We aimed to target normoxia and normocapnia using lung protective ventilation parameters since the first day of ICU admission (PEEP > 5, tidal volume 6–8 ml/kg, plateau pressure < 30cmH_2_O and mostly volume control mode to stabilize the PaCO_2_). Management of intracranial pressure was classified according to the therapy intensity level (TIL) [21].

Age less than 18 years old and pregnancy were exclusion criteria. Protocol and anonymized data collection were approved by the local research Ethics Committee.

### Patient and Monitoring Data

Clinical records were revised for age, gender, length of stay (LOS) at the NCCU and hospital and mortality rates at hospital discharge. Patients were classified according to general severity scores such as Glasgow Coma Scale at hospital admission, APACHE II (Acute Physiology And Chronic Health Evaluation II) [22] and Marshall classification of CT-scan [23] for TBI. Outcome at three months was evaluated with Glasgow Outcome Score [24].

During the first 10 days after admission, we collected the parameters of the first blood gas analysis of each day (PaO_2_, PaCO_2_, SaO_2_, pH, HCO_3_, FiO_2_, PFratio and shunt fraction as Qs/Qt) and the correspondent ventilator data (ventilation mode, respiratory rate (RR), minute ventilation (MV), plateau pressure (Ppl), positive end-expiratory pressure (PEEP), lung compliance and driving pressure calculated as the difference between plateau pressure and PEEP (ΔP=Ppl-PEEP)). Systemic and brain monitoring data were calculated as daily mean values, including arterial blood pressure (ABP), heart rate (HR), intracranial pressure (ICP), CPP, PRx, CPPopt and the difference between CPP and CPPopt (CPP-CPPopt). ABP was measured and calibrated at heart level and patients were treated with 30° head-up elevation [25]. We measured two indices related to increased risk of mortality: the percentage of total time of monitoring spent with impaired autoregulation, defined by PRx > 0.25 and the percentage of time spent with inadequate CPP, defined as CPP < CPPopt [17,20]. We also calculated the dose as time (hour) plus unit values for both risk variables, namely % of total time without autoregulation and % of total time with CPP < CPPopt. Acute lung injury was documented with chest imagiology, namely X-ray and CT-scan, if indicated.

### Statistical analysis

Demographic data analysis of continuous variables were summarized by mean values and standard deviations (mean ± SD) or medians and interquartile ranges (median; (IQR)), according to the symmetry of the distribution. The discrete variables were presented as a count or percentage. Longitudinal data related to evolution and outcome of the patients along time were analyzed by a linear mixed-effects regression model, taking into account the within-subject variability [26]. The random effect was identified only at the intercept for the single grouping level (subjects). For the identification of the best model, regressions with different fixed linear predictors, random effects structures, residual correlation matrixes and residual variances were considered. Comparison between models was based on the likelihood ratio test for nested models and on the Bayesian Information Criteria (BIC) otherwise.

Statistical analysis was performed with the R language and software environment for statistical computation, version 3.3.2 [27]. Statistical significance was set at 0.05.

## RESULTS

### Descriptive data

We revised 139 clinical cases of adult trauma patients with severe TBI admitted to the NCCU in the studied period and rejected 47 cases due to incomplete clinical or monitoring data. We performed the final analysis of 92 cases, 79 (86%) male and with a mean age of 53±21 years old. Multiple trauma with TBI was presented in 45 (49%) patients and 19 (21%) had thoracic trauma.

At hospital admission, the median GCS was 7 (IQR 5) and all mild TBI patients included presented early deterioration to GCS < 8. Regarding other severity scores, the median CT Marshall Classification was 3 (IQR 2) and mean APACHE II was 19±6 with a mean predicted hospital mortality of 33±17%, but the real hospital mortality rate was 15.2% (14 deaths: 5% brain death and the remainder were related to withdrawal of care).

During the first 10 days after NCCU admission mean values for lung monitoring variables were PFratio of 305±88 with FiO_2_ of 0.5±0.13, which corresponded to mean shunt fraction (Qs/Qt) of 16.3±6.7 %, Ppl 19.7±4.8 cmH_2_O, ΔP 15.9±3.5 cmH_2_O and lung compliance 43±13.8 ml/cmH_2_O. At day 0, there was no significant difference in mean values of PFratio (p=0.544) and Ppl-PEEP (p=0.520) related to outcome (dead or alive). Mean daily fluid balance was 171±564 ml/day and the burden of disease evaluated with the score Therapy Intensity Level (TIL) had a median of 2 (IQR 1). We found no correlation between fluid balance and PFratio.

The the most relevant averages for systemic and brain monitoring data were ICP 11.2±5.8 mmHg, CPP 85.9±7.4 mmHg, PRx 0.03±0.19, CPPopt 88.7±8.5 mmHg, CPP-CPPopt −2.8±10.2 mmHg. The percentage of total time of monitoring spent with impaired autoregulation (PRx>0.25) was 29.3±19.4% and within the critical region of hypoperfusion (CPP<CPPopt) was 56.3±27.5%. Median GOS at 3 months was 3 (IQR 2).

**Fig. 2.**
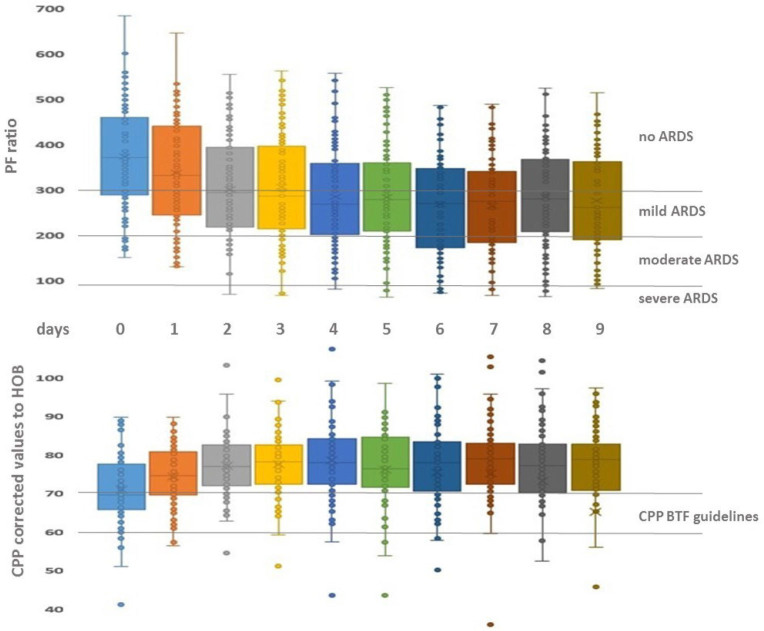
**Time evolution of PF ratio across the first ten days of admission for the whole sample according to ARDS definition of PFratio intervals by the Berlin Task Force (top). CPP evolution across the first ten days of admission for the whole sample and CPP definition interval according to Brain Trauma Foundation (bottom).** PF ratio: ratio between oxygen arterial pressure and inspired fraction of oxygen; CPP: Cerebral Perfusion Pressure; HOB: Head of bed elevation (30^o^).

All other demographic, monitoring and management data are described in Table 1 and Figure 2.

**Table 1.** Demographic, Monitoring, Management and Outcome data of patients with severe acute Traumatic Brain Injury.

Variables		Mean±sd /Median (IQR)
**Demographic Data**		
Number of Patients	Total	92
	Multiple Trauma	45 (49%)
	Thoracic Trauma	19 (21%)
Age (Years)		53 ± 21
Gender	Male	79 (86%)
	Female	13 (14%)
GCS at admission		7 (IQR 5)
APACHE II		19 ± 6
Apache II mortality (%)		33 ± 17
CT Marshall Classification		3 (IQR 2)
**Monitoring and Management Data**		
FiO_2_		0.5 ± 0.13
PFratio		305 ± 88
PaO_2_(mmHg)		146.5 ± 28.5
PaCO_2_(mmHg)		38.1 ± 3.7
SaO_2_(%)		98.6 ± 0.74
RR (cycles/min)		17 ± 4
MV (L/min)		9.45 ± 2.18
PEEP (cmH_2_0)		6,2 ± 1,3
Ppl (cmH_2_0)		19.7 ± 4.8
DP=Ppl-PEEP (cmH_2_0)		15.9 ± 3.5
Shunt fraction (Qs/Qt) (%)		16.3±6.7
Compliance		43 ± 13.8
Fluid Balance (ml/d)		171 ± 564
Therapy Intensity Level (TIL)		2 (IQR 1)
HR (bpm)		71.9 ± 10.5
ABP (mmHg)		96.7 ± 7.0
ICP (mmHg)		11.2 ± 5.8
CPP(mmHg)		85.9 ± 7.4
PRx		0.03 ± 0.19
CPPopt (mmHg)		88.7 ± 8.5
CPP-CPPopt (mmHg)		−2.8 ± 10.2
**Outcome Data**		
LOS ICU (days)		22 ± 26
LOS Hosp (days)		48 ± 48
Mortality		14 (15.2%)
GOS at 3 months		3 (IQR 2)

sd – standard deviation; IQR – interquartile range; GCS-Glasgow Coma Scale; APACHE II - Simplified Acute Physiology Score; APACHE II % - mortality prediction at hospital discharge; CT-Marshall Classification (CT classification for TBI). FiO_2_ (Fraction of inspired O_2_,), PFratio (PaO_2_/FiO_2_ ratio), PaO_2_ (arterial oxygen pressure), PaCO_2_ (arterial carbon dioxide pressure), SaO2 (% of saturation of O_2_), RR (respiratory rate), MV (respiratory minute *volume), PEEP (positive end-expiratory pressure)*, Ppl (plateau pressure), DP=Ppl–Peep (driving pressure), Compliance, HR (heart rate), ABP (mean arterial blood pressure), ICP (intracranial pressure), CPP (cerebral perfusion pressure, PRx (pressure reactivity index), CPPopt (optimal CPP), CPP-CPPopt (difference between CPP and CPPopt), LOS ICU/hosp (length of stay in intensive care unit / in hospital), GOS at 3M (Glasgow outcome scale at 3 months; 1 - dead).

### Longitudinal comparative data

The results from the regression analyses are presented in Table 2. In the model for the PFratio, the random effect at the intercept (day 0) accounts for 38.9 % of the total data variability. The model shows that the PFratio has a significant quadratic dependence on time (days) with positive curvature. The lowest PFratio is predicted to happen around the 6th day after hospital admission. Women are expected to have a PFratio greater than men by 99 units, on average. The variables PRx and driving pressure (ΔP=Ppl-PEEP)) are negatively and significantly associated with PFratio. An increase of 0.1 units in PRx is predicted to lower PFratio, on average, by 5.0 as well as each unit increase in ΔP is predicted to lower PFratio by 5.3. Both CPP and CPPopt were not found to have a statistically significant effect on PFratio (p=0.533 and p=0.556, respectively).

**Table 2.** Estimates from the final mixed-effects regression models for PFratio, CPP-CPPopt and Driving Pressure across time (10 days), adjusted for other variables of interest.

Variables	FIXED EFFECTS			RANDOM EFFECT
	Coefficient	St Error	p-value	St deviation
**Model for the time-effect on *PFratio***				
Intercept	436.42	17.63	<0.001	59.25
Time (days)	−27.12	4.56	<0.001	
Days^2^	2.175	0.48	<0.001	
Female	99.28	23.39	<0.001	
PRx	−49.99	15.68	0.002	
Ppl-Peep	−5.34	0.97	<0.001	
**Model for the time-effect of outcome on *CPP-CPPopt***				
Intercept	−2.37	1.58	0.133	2.95
Dead	−0.79	1.72	0.647	
Time (days)	−0.56	0.28	0.048	
Time*Dead	1.10	0.31	<0.001	
**Model for the time-effect of outcome on** *Driving Pressure*				
Intercept	14.76	1.10	<0.001	2.85
Dead	−0.43	1.20	0.718	
Time (days)	0.68	0.15	<0.001	
Time*Dead	−0.34	0.17	0.041	

For the PFratio model, the best structure for the residual correlation matrix was a time autocorrelation structure of order 1, with a coefficient estimated at 0.384 (95% CI: 0.272 – 0.485). The model evaluating the time-effect of the outcome (Dead/Alive) on CPP-CPPopt and driving pressure used the same residual correlation structure and the coefficient was, respectively, estimated at 0.231 (95% CI: 0.123 - 0.334) and 0.426(95% CI: 0.312 – 0.527). Female (sex), PRx (pressure reactivity index), Ppl–Peep (driving pressure as the difference between plateau and Peep pressures).

**Fig. 3.**
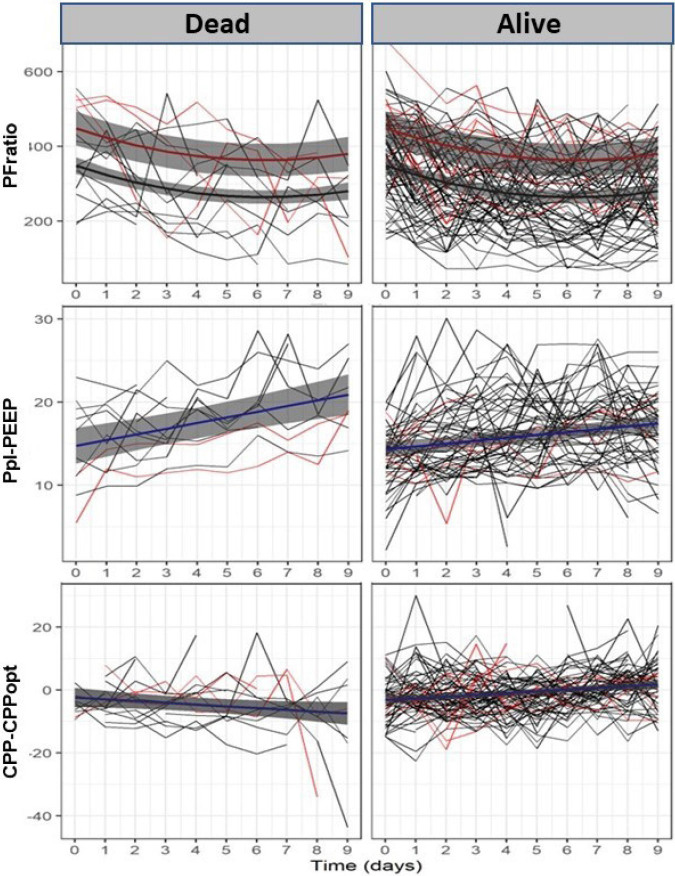
**Time evolution of PFratio (top), Ppl-PEEP (middle) and CPP-CPPopt (bottom), for each patient (fine lines), according to the Outcome (Dead or Alive) and Sex (Female, in red; Male, in black). The mean and 95% confidence band of the prediction model, based on the fixed-effects, are also pictured.** PFratio: ratio between oxygen arterial pressure and inspired fraction of oxygen; Ppl–PEEP: difference between plateau pressure and PEEP (driving pressure); CPP-CPPopt: difference between cerebral perfusion pressure and optimal CPP.

In addition, a significant positive association between outcome and CPP-CPPopt interval was found (Table 2). A linear mixed-effects regression model evaluating the time effect of outcome on CPP-CPPopt identified opposite time effects for the two classes. While, at day 0, CPP-CPPopt is not significantly different between dead and alive, as time evolves the model expects: (1) alive individuals to significantly increase CPP-CPPopt on average by 0.5 mmHg each day; (2) dead individuals to progressively lower their CPP-CPPopt values, at a rate of 0.6 mmHg per day (p=0.048). The model estimates that 16.6 % of the total variability in the data is due to inter-individuals variability. Figure 3 represents mean and 95% confidence bands of the prediction model for each response variable, based on the fixed-effects.

A similar model was considered for the driving pressure, studying the longitudinal effect of outcome (Table 2). While, at day 0, there are no significant differences in ΔP between dead and alive, their trajectories over time then become significantly different from one another. The model estimates dead individuals to significantly increase ΔP 0.684 cmH_2_O on each day (p<0.001) while it estimates alive individuals to increase by 0.345 cmH_2_O on each day (p=0.041). The random effect at the intercept accounts for 43.7% of the total data variability.

## DISCUSSION

Adequate management of CPP remains a key problem for best care in Traumatic Brain Injury [12]. In fact, CPP target should be sufficient to ensure perfusion in the range of autoregulation in order to provide a stable cerebral blood flow, without triggering systemic detrimental effects [28,29].

In our study, we confirmed that estimates of CPPopt and real CPP measured at heart level were around 15 mmHg higher compared to BTF CPP recommendation [11]. However, the clinical interpretation for this CPP divergence should be corrected for the level of ABP calibration and head-up elevation, as described in material and methods section and showed in figure 2 [25]. This corrected CPP value (similar to BTF CPP threshold) may be one of the reasons why, although individual PFratio deteriorates across time, both CPP and CPPopt were not found to have a statistically significant effect on PFratio between individuals. In recent publications, Thiara et al. (2018) and Moreira et al. (2018), CPP and CPPopt have not been showing to be associated with PFratio deterioration and consequently to increase the risk of development of lung injury and ARDS as defined by the Berlin definition [7,29,30]. In our TBI population, there was no statistically significant difference at the first day, for lung and oxygenation conditions according to PFratio and ΔP, despite 21% of patients had thoracic trauma.

Our results are consistent with previous studies that relate an association between the increase of driving pressure and PFratio worsening as well as its significant association with outcome as published by Amato *et al* (2015) [31]. In his work, he stated that not only driving pressure was the best variable to stratify the risk of ARDS but was also strongly associated with increased survival. Likewise, in paper from Tejerina *et al* (2017) and Thiara *et al* (2018) increased driving pressure was associated with the development of ARDS [29,32].

Curiously, we found that women are expected to have a PFratio greater than men, although with no statistically significant difference in outcome, as shown by Yeung *et al* (2011), which compared outcome in male and female in age groups after traumatic brain injury and did not find a significant association between gender and mortality [33].

In our study, we were also able to demonstrate that an increase in PRx was negatively associated with PFratio variation along time. This raises the question of a possible association between impairment of cerebral autoregulation and lung injury. It is known that severe TBI may induce lung distress and edema, such as neurogenic pulmonary edema [5,34].

Consistently, our results showed that there was a significant positive association between outcome and CP-Popt-target management. In fact, the individuals that have died spent a longer time with a higher negative difference between CPP and CPPopt (CPP-CPPopt), compared to the ones who have survived. These data are in agreement with the published results both from retrospective center series and prospective clinical protocols analysis [35,36].

Finally, our longitudinal analysis and predictive model confirms the importance of time in the natural course of brain-lung injury and brain-lung interactions and showed that therapeutic strategies that protect the brain such as CPPopt-guided therapy may also avoid lung dysfunction [37].

There are limitations to our study. First, this a single-center retrospective study with a limited sample size. In addition, we presented a pathophysiological approach of lung injury and ARDS as defined by the Berlin definition and therefore we did not analyzed ancillary variables for severe ARDS, such as chest x-ray severity. Lastly, we gave privilege to study the effect of time on PFratio and consequently, due to this longitudinal model, we preferred not to define different severity risk groups of acute respiratory distress syndrome. Nevertheless, we analysed oxygenation status and performed statistical adjustment of the dataset, which corroborated no clinical differences in oxygenation and lung parameters at NCCU admission.

## CONCLUSION

In conclusion, severe TBI patients managed with individualized CPPopt-guided protocol presented better outcome without association with the development of acute lung injury. However, increasing driving pressure along time, increased the risk of developing lung injury.
